# Intention to Engage in Mountain Sport During the Summer Season in Climate Change Affected Environments

**DOI:** 10.3389/fpubh.2022.828405

**Published:** 2022-07-07

**Authors:** Martin Niedermeier, Anika Frühauf, Martin Kopp

**Affiliations:** Department of Sport Science, University of Innsbruck, Innsbruck, Austria

**Keywords:** climate change, mountain hiking, glacial shrinkage, affective responses, behavior, Theory of Planned Behavior

## Abstract

Natural environments can make it easier to engage in regular physical activity, including mountain sport activities. However, global warming is expected to change natural environments, especially in mountainous regions with potential impacts on physical activity behavior. While there is some evidence of a reduced intention to engage in winter sport in climate change affected environments, little is known on the impact of climate change in mountain sports conducted in the summer season. Therefore, the present study aimed at comparing the effect of being exposed to a climate change affected scenario (CCA) to being exposed to a climate change unaffected scenario (CCU) on the intention to engage in summer mountain sport activities. Furthermore, we aimed to analyze the role of anticipated affective responses in the context of the Theory of Planned Behavior (TPB). Using a web-based experimental cross-sectional study design, participants were randomly allocated to scenarios of either CCA or CCU pictures. Participants were asked to complete questions about TPB variables and about affective responses referring to the displayed scenarios. Statistical analyses included tests on group differences and hierarchical linear regression analyses. TPB variables (intention to engage in summer mountain sport, attitude, and perceived behavioral control) did not show significant group differences between CCA (*n* = 155) and CCU (*n* = 156), *p* > 0.131; *r* < −0.10. Significantly lower anticipated affective valence was found in CCA compared to CCU, *p* < 0.001, *r* = −0.43. Affective valence did not significantly improve the TPB model, change in *R*^2^ = 0.7%, *p* = 0.096. However, a higher affective valence was significantly associated with a higher attitude toward summer mountain sport, beta = 0.19, *p* < 0.001. Intention to engage in summer mountain sport was similar in the groups. Therefore, an immediate reduced engagement in mountain sport activities due to climate change seems unlikely in the summer season, although differentiated findings across various activities of summer mountain sport cannot be excluded. A reduced affective valence during summer mountain sport activities might occur in the presence of signs of climate change in the environment, which may lead to longer-term behavior changes in climate change affected scenarios also in the summer by repeated experiences of reduced valence.

## Introduction

Physical inactivity is a major public health issue and a leading risk factor for the development of non-communicable diseases such as cardiovascular diseases and diabetes (1). Participating in physical activity can help in the treatment of diseases (2). Therefore, the World Health Organization has set the goal of reducing physical inactivity by 15% by the year 2030 (3). However, data do not show significant changes in physical activity patterns between 2001 and 2016 (1).

The regularity of being physically active is influenced by a variety of factors including (a) behaviorally relevant cognitive variables (4), (b) affective responses to previous physical activity experiences (5), and (c) characteristics of the environment where physical activity takes place. Referring to (a), the Theory of Planned Behavior (TPB) (4) or the Reasoned Action Approach (6) have commonly been used in the development of health related intervention strategies to evaluate a certain predicted future behavior (7, 8). TPB is also used in the context of physical activity behavior and provides important cognitive-based determinants of intention to engage in physical activity: According to TPB, behavior (e.g., engaging in physical activity) is determined to a large amount by behavioral intentions (i.e., readiness to engage in physical activity). Behavioral intentions in turn are based on subjective norm, attitude, and perceived behavioral control (4). Referring to (b), there is evidence that positive affective valence during and after physical activity is associated with a higher level of future physical activity (9, 10). Furthermore, affective responses might explain variations in the regularity of being physically active dependent on certain characteristics of the physical activity [e.g., mode or intensity (11)]. Referring to the environmental aspects mentioned in (c), natural spaces seem to play an important role for physical activity behavior. For instance, higher amounts of natural space in the living area seem to be associated with undertaking more (and potentially more vigorous) physical activity, e.g., cycling or walking (12). Additionally, there is evidence that a higher likelihood of meeting physical activity guidelines (i.e., achieving at least 600 metabolic equivalent task minutes per week) is positively associated with visit frequency to local green spaces (13). The environment, where physical activity takes place, may also interact with the factors influencing physical activity behavior. In this context, synergistic benefits of physical activity and the environment on wellbeing are discussed: Shanahan et al. hypothesize positive effects on wellbeing when being physically active in natural spaces that are larger than the independent effects of being active (not in a natural environment) and the natural environment (not being physically active). The better wellbeing might increase physical activity behavior in the long-term (12). There is evidence of a better wellbeing/more positive affective responses during and after physical activity bouts in natural environments compared to indoor physical activity (14–16). Furthermore, environmental proximity to recreation is discussed in the context of the Theory of Planned Behavior variables (17).

Climate change influences natural environments (18) and may also reduce physical activity level in these areas. Some areas are more prone to rapid and visible consequences of climate changes, such as alpine environments (19). Glacier retreat, less snowfall at lower altitudes, and changes in the snow line are consequences connected with climate change in alpine environments (18, 20). Climate change can also affect physical activity patterns, especially, when the physical activity takes place outdoors. In an earlier study, we found a reduced intention to engage in recreational winter sport activities, such as alpine skiing or snowboarding, when being exposed to a winter sport scenario showing an environment affected by climate change (16). Therefore, climate change might be associated with a reduced winter sport activity or with modifications in the winter sport habits (e.g., going to ski resorts at higher altitude). The results further indicated that—consistent to behavior theories—both cognitive variables of the Theory of Planned Behavior (4), such as attitude, and non-cognitive variables (9, 10), specifically affective valence were relevant variables to be considered in the intention to engage in winter sport activities (16).

However, potential effects of climate change on summer sport remain elusive although summer mountain sport plays a similarly high economical role as winter sport according to the bed occupancy rate in Austria (winter 2018/19: 38% and summer 2019: 37%) (21). Furthermore, the direction of the effects, i.e., an increase or a decrease of physical activity behavior, is unclear. In the United States, climate change was predicted to negatively impact frequency of hiking (by 20% in the year 2060), but to show a positive effect on the frequency of swimming or horseback riding (22). Similarly, given the variety of different mountain sport activities in mountainous regions during the summer season (23), the effect of climate change on overall physical activity in these regions might be unclear.

Following these considerations, the aim of the present study was 2-fold: Aim 1 was to analyze the effect of exposure to a climate change affected scenario on the intention to engage in recreational summer mountain sport activities in comparison to a climate change unaffected scenario. Attitude, perceived behavioral control, and anticipated affective responses during engagement in the different scenarios were also compared. Aim 2 was to analyze the role of anticipated affective responses in the intention to engage in mountain sport in the context of TPB.

## Materials and Methods

### Design and Sample

**Figure 1:**
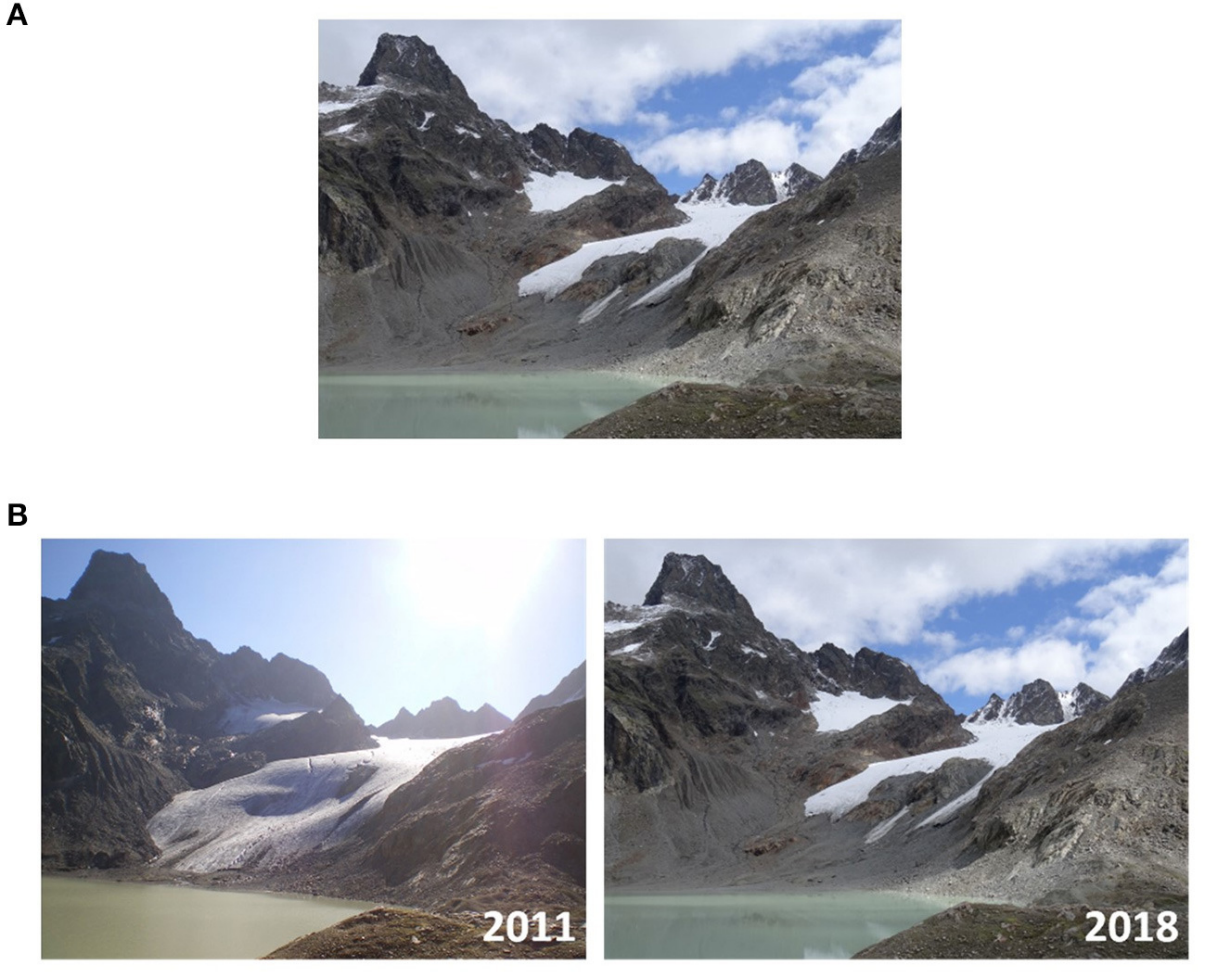
Climate change unaffected scenario [**(A)**, upper row] and climate change affected scenario [**(B)**, lower row]. Pictures by courtesy and with kind permission of Austrian Alpine Association.

A web-based questionnaire was distributed by the University of Innsbruck's Email announcement and by the national alpine club (“Austrian Alpine Association”) via social media announcement. The calculation of the exact number of recipients remains elusive since it is possible to unsubscribe from the University's Email announcement and social media range of the Austrian Alpine Association remains vague. The procedure was widely similar to our previous study (16): A short information at the start of the online survey was given that the study focuses on motivational aspects in recreational mountain sport practiced in the summer season. No information about climate change was added in the initial information. During completion of the questionnaire, participants were randomly allocated (simple randomization) to one of two groups: (1) A group receiving two pictures—one from 2011 with larger glacier and one of the same place from 2018 with smaller glacier—indicating a climate change affected scenario (CCA group) and (2) a group receiving the picture of 2018 only without mentioning the year (CCU group, Figure 1). The pictures pertaining the scenarios were placed at the top of the pages asking participants to look closely at the scenarios and to answer the following questions referring to the corresponding scenario.

Inclusion criteria for participation were given at the recruitment announcement: (1) Conducting mountain sport activities in the summer season 2021, and (2) aged at least 18 years. The questionnaire was provided in German language only. No incentives were provided for participating. The study procedure was approved by the Board for Ethical Questions in Science of the University of Innsbruck in accordance with the Declaration of Helsinki (#44/2021, date: 21.06.2021).

### Questionnaire

Using a total of 31 questions, information about socio-demographic and summer mountain sport characteristics, affective responses, TPB variables, and future summer mountain sport engagement. Time to complete the questionnaire was ~10 min.

### Measurements

#### TPB Variables

Behavioral intentions, attitude toward behavior and perceived behavioral control were assessed following the TPB guidelines by Ajzen (24). All questions asked in Frühauf et al. (16) were adapted from winter mountain sport to summer mountain sport and were answered on a seven-point Likert scale. On each page the participants were reminded to answer the question with reference to summer mountain sport participation in the displayed scenario. Intention to engage in summer mountain sport was assessed with three items (e.g., I intend to participate regularly in mountain sport activities in the upcoming summer season). Cronbach's alpha for intention in the present sample was 0.92. Five items with two opposing attributes as anchors at the poles (e.g., harmful/beneficial) were used to assess attitude toward summer mountain sport (Cronbach's alpha = 0.85). Perceived behavioral control was evaluated using four items (e.g., I am confident that I will be able to regularly conduct mountain sport activities in the coming summer season. Cronbach's alpha = 0.87). After recoding the negatively formulated items for attitude, the mean of all items belonging to each TPB variable was calculated by adding the values of the TPB variables belonging to one subscale and dividing the sum by the number of items. Therefore, the values for all TPB variables ranged from 1 (low intention, attitude, perceived behavioral control) to 7 (high intention, attitude, perceived behavioral control). Based on our previous findings (16), where the dimension of subjective norm was less relevant in the context of winter mountain sport intention, we did not assess subjective norm.

#### Future Summer Mountain Sport Engagement

Future summer mountain sport engagement in case of large glacier shrinkage was assessed based on Pickering et al. (25), who studied attitudes of skiers to climate change in Australia. Pickering et al. (25) used a single item question (“Assuming that the next five winters had very little natural snow: please circle one option as to where you would ski/snowboard”) with four response options (“Australia same frequency,” “Australia less often,” “Overseas” or “Give up”). We adapted the question and response option to meet the characteristics of summer mountain sport: We included an additional response option for the possibility to increase mountain sport frequency. The response options ranged from “Quit mountain sport,” “Conduct mountain sport further away,” “Conduct mountain sport less often than now,” “Conduct mountain sport in the same frequency as now,” and “Conduct mountain sport more often than now.” Given the variety of summer mountain sport activities (26), a separate question referring to future summer mountain sport engagement in case of large glacier shrinkage was posed for each summer mountain sport activity.

Potential consequences of climate change on mountainous regions were selected in accordance to previous literature (27). The participants were asked if and how these consequences change their engagement in summer mountain sport activity. Using a five-point Likert scale, a bipolar response mode was chosen ranging from “Quit mountain sport,” over “Less regularly,” “Does not concern,” “More regularly,” to “Much more regularly.”

#### Affective Responses

Affective responses were assessed when the pictures on the scenarios appeared for the first time. The participants were instructed as follows: “We ask you to first observe the picture(s) and then answer the questions below in the imagination of engaging in mountain sport activities in the environment seen in the picture(s).” The basic dimensions of affective valence and perceived activation were assessed with two single-items measures based on the Circumplex Model (28). Affective valence was assessed using the German version (29) of the Feeling Scale (30) with 11 response options ranging from “Very good” (+5) to “Very bad” (−5). The German version of the Felt Arousal Scale ranging from 1 (“low arousal”) to 6 (“high arousal”) was used to assess perceived activation (31). Information on validity of the two scales for the German version can be found in Maibach et al. (29).

#### Additional Variables

Information on summer mountain sport participation of the participants was collected asking for the regularity of participation in eight summer mountain sport activities with possible answers ranging from 1 (never) to 7 (regularly). Physical activity level not related to mountain sport was assessed with two questions asking about physical activity during leisure time and at work (32). Consequently, a physical activity level indicator ranging from 1.4 (low physical activity level) to 2.3 (high physical activity level) and an energy expenditure estimator (expressed in MJ) can be calculated. The energy expenditure estimator considers the physical activity level, weight status, age group, and sex. A short version of the risk-perception scale with three items and four response options (33) was used to assess risk-perception of climate change. The scale uses the mean of the three items to express risk-perception of climate change with 1 (low perceived risk of climate change) to 4 (high perceived risk of climate change). Cronbach's alpha of the present sample was 0.74.

### Statistical Analyses

Statistical analyses for aim 1 consisted of tests on group differences between CCA and CCU group using SPSS v. 27 (IBM, New York, NY, USA). Differences in TPB variables (intention to engage in summer mountain sport, attitude toward summer mountain sport, and perceived behavioral control) and anticipated affective responses during summer mountain sport (affective valence and perceived activation) were analyzed with the Mann-Whitney *U*-Test due to non-normality of data (tested by the Shapiro-Wilk test for each subgroup). Rosenthal's *r* was calculated as an effect size where Mann-Whitney *U*-Test was used with the conventions small (0.1), medium (0.3), large (0.5) (34) Differences in future summer mountain sport engagement in case of large glacier shrinkage were analyzed using Pearson's χ^2^ tests. To meet the assumptions for future summer mountain sport engagement in case of large glacier shrinkage, the lowest three response options were combined resulting in a comparison of three response options only. Since the effects of climate change might be most severe in mountaineering sports (27), a subsample analysis of group differences between CCA and CCU group was done in mountaineers only. Participants who stated to engage in mountaineering more than never were considered as the subsample of mountaineers.

For aim 2, a series of linear regression analyses were performed using jamovi v. 2.0 (35). Attitude was modeled as an outcome of group allocation or affective valence. We abstained from an a priori planned mediation analysis of the outcome attitude (16) since group allocation was not significantly associated with attitude. Furthermore, a hierarchical regression analysis with two models was used to model intention to engage in summer mountain sport as an outcome of attitude, perceived behavioral control (model 1), and affective valence (model 2). *R*^2^ of model 1 and model 2 was compared to analyze if the inclusion of affective valence significantly improved the model. *P*-values <0.05 were considered as significant. Unless otherwise stated, values represent mean (SD), Median (interquartile range), and relative (absolute) frequencies.

## Results

### Sample Characteristics

In total, *n* = 311 participants completed the questionnaire, out of which 49.8% (*n* = 155) were allocated to the CCA group and 50.2% (*n* = 156) to the CCU group. Sample characteristics including physical activity behavior, risk perception of climate change, and summer mountain sports activities were relatively similar in both groups (Table 1). The majority of the sample was regularly engaging in mountain hiking. Trail running was a not-listed summer mountain sport mentioned by 7.7% (*n* = 24) of the participants.

**Table 1:** Descriptive information on demographic variables, physical activity, risk-perception of climate change, and summer mountain sport activities of the total sample and by group.

	**Total sample** **(*****n*** **=** **311)**		**Climate change unaffected scenario** **(*****n*** **=** **156)**		**Climate change affected scenario** **(*****n*** **=** **155)**	
	**Mean**	**(SD)**	**Mean**	**(SD)**	**Mean**	**(SD)**
Age (years)	27.9	(10.4)	27.3	(9.5)	28.5	(11.3)
Body mass index (kg/m^2^)^a^	21.9	(3.5)	21.7	(3.8)	22.2	(3.2)
Physical activity level (1.4: low, 2.3: high)^a^	1.9	(0.2)	1.9	(0.2)	1.8	(0.2)
Energy expenditure (MJ)^a^	11.8	(2.6)	11.7	(2.7)	11.8	(2.5)
Risk-perception index of climate change (1: low, 4: high)	3.1	(0.7)	3.0	(0.7)	3.3	(0.6)
Frequency of summer mountain sport activities (n/month in the summer season) ^a^	8.3	(6.4)	8.0	(5.9)	8.7	(6.9)
	%	(*n*)	%	(*n*)	%	(*n*)
**Sex** ^**a**^
Female	55.9	(174)	53.2	(83)	58.7	(91)
Male	42.8	(133)	44.9	(70)	40.6	(63)
**Regular summer mountain sport activity (>** **4 out of 7)**
Mountain hiking on wider trails, valley paths or forest paths	62.4	(194)	60.3	(94)	64.5	(100)
Mountain hiking on narrower mountain paths or climbs	81.7	(254)	79.5	(124)	83.9	(130)
Mountain biking	30.5	(95)	26.9	(42)	34.2	(53)
Fixed-rope climbing (via ferrata)	17.4	(54)	17.9	(28)	16.8	(26)
Outdoor sport climbing	18.0	(56)	16.7	(26)	19.4	(30)
Outdoor bouldering	4.8	(15)	5.1	(8)	4.5	(7)
Alpine multipitch climbing	10.3	(32)	12.2	(19)	8.4	(13)
Mountaineering (glacier)	22.8	(71)	25.6	(40)	20.0	(31)

*^a^missing data: n <14*.

### Group Differences

None of the TPB variables were rated significantly different between groups (Table 2). Intention to engage in summer mountain sport, attitude toward summer mountain sport, and perceived behavioral control were rated similarly in the CCA and CCU group. In contrast, anticipated affective valence and perceived activation were significantly lower in the CCA group compared to the CCU group.

**Table 2:** Theory of Planned Behavior variables, affective responses, and future summer mountain sport activity engagement by group.

	**Climate change unaffected scenario** **(*****n*** **=** **156)**				**Climate change affected scenario** **(*****n*** **=** **155)**				***Z***	***P***	***r***
	**Mean**	**(SD)**	**Med**	**(IQR)**	**Mean**	**(SD)**	**Med**	**(IQR)**			
**Theory of planned behavior variables (1: low, 7: high)**											
Intention to engage in summer mountain sport	6.5	(1.0)	7.0	(6.3–7.0)	6.6	(0.8)	7.0	(6.3–7.0)	−1.16	0.245	−0.07
Attitude toward summer mountain sport ^a^	6.5	(0.8)	6.8	(6.2–7.0)	6.3	(1.0)	6.6	(6.0–7.0)	−1.51	0.132	−0.09
Perceived behavioral control	6.3	(1.0)	6.5	(6.0–7.0)	6.2	(1.3)	6.8	(6.0–7.0)	−0.09	0.930	−0.01
**Affective responses during summer mountain sport**											
Affective valence^a^	3.0	(2.3)	4.0	(2.0–5.0)	0.5	(3.0)	0.0	(−1.0–3.0)	−7.58	**<0.001**	–**0.43**
Arousal^a^	5.0	(1.2)	5.0	(4.0–6.0)	4.5	(1.4)	5.0	(4.0–6.0)	−3.22	**0.001**	–**0.18**

*^a^missing data: n <6, Med: Median, IQR: interquartile range, bold values indicate significant group differences*.

When the analysis was limited to the subsample of mountaineers (*n* = 155), no significant differences in the TPB variables were found, *z* < 0.96, *p* > 0.337. Similar to the total sample, affective valence and perceived activation during imagination of summer mountain sport were significantly lower in the CCA group compared to the CCU group, *z* > 3.34, *p* < 0.001.

**Figure 2:**
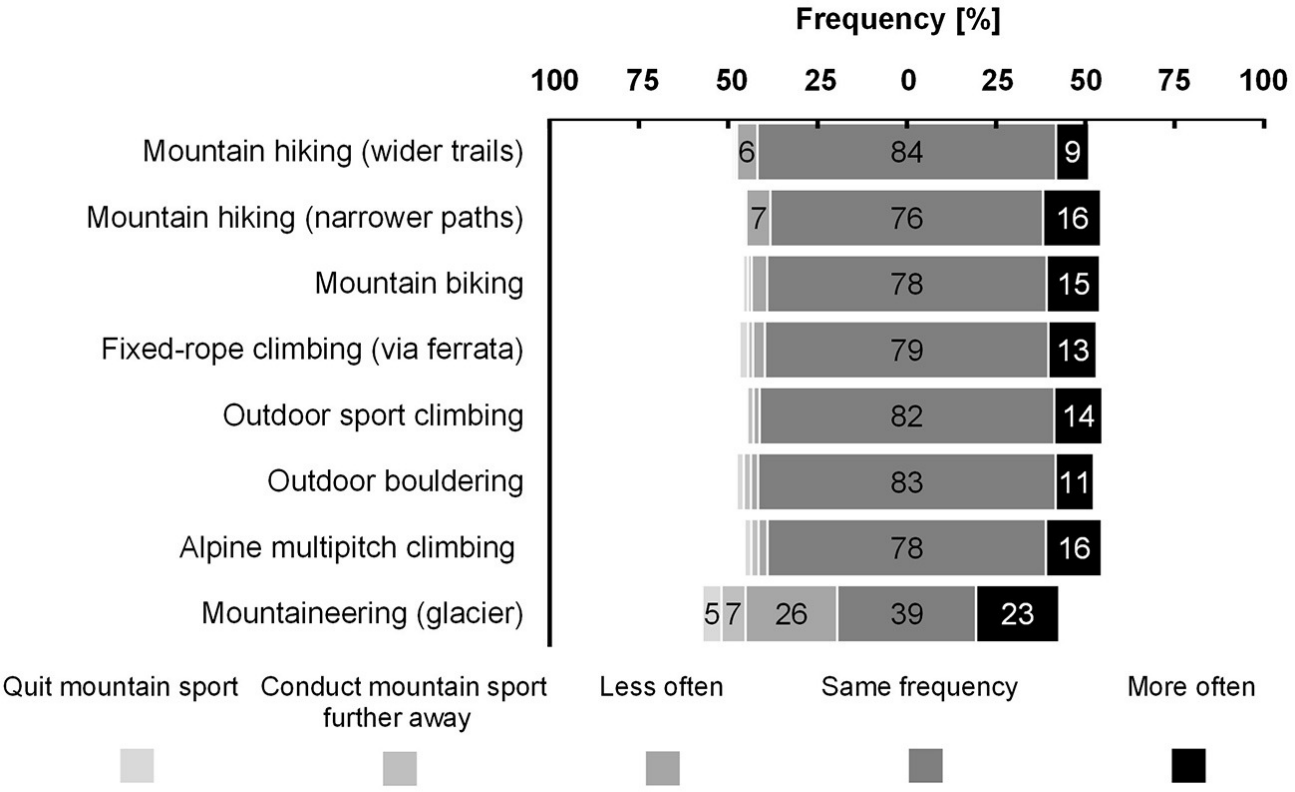
Future summer mountain sport engagement in case of large glacier shrinkage of the total sample. No significant differences were found between groups, *p* > 0.085. Values below 5% were not specified.

The responses in the categories of future summer mountain sport engagement in case of large glacier shrinkage were not significantly different between groups, χ^2^ (2, *N* > 149) <4.92, *p* > 0.085. The majority of the total sample responded to conduct their summer mountain sport in the same frequency as at present in most of the sports and between 9 and 23% reported to increase the frequency (Figure 2). Mountaineering contained the largest proportion of a reduced frequency or conducting the mountain sport further away (38%) in comparison to the other summer mountain sports (<8%).

**Figure 3:**
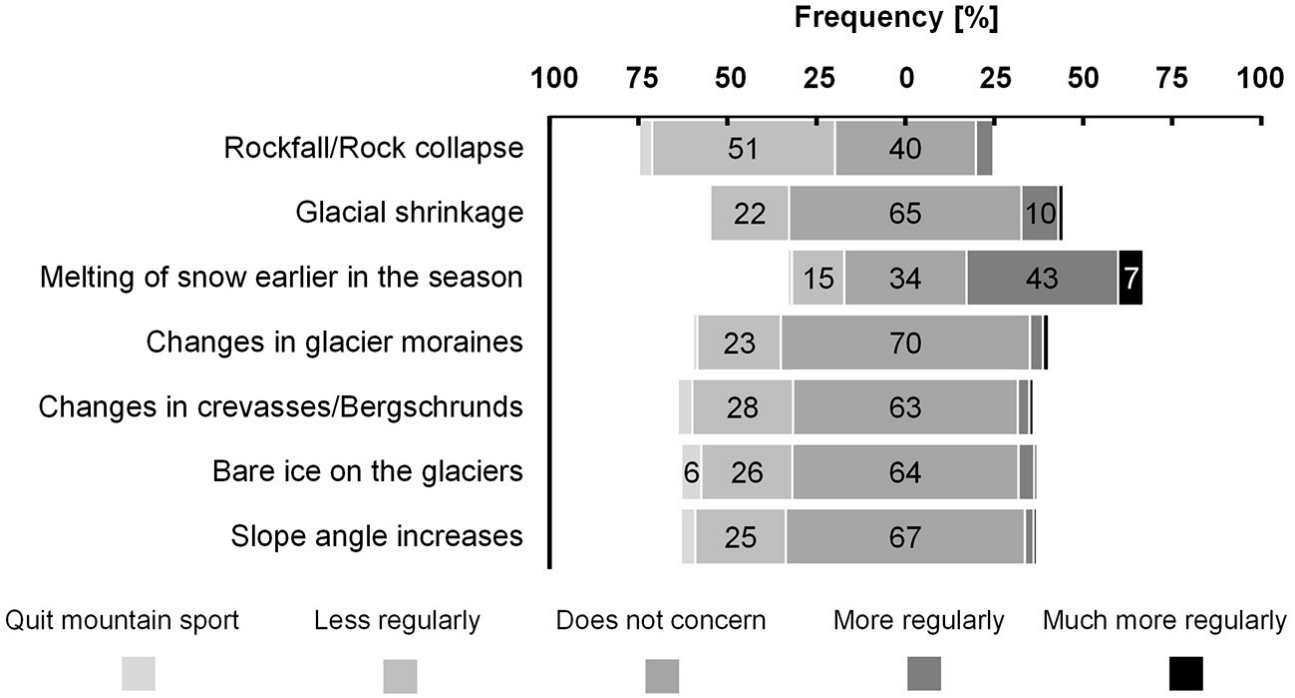
Engagement in summer mountain sport when being confronted with consequences of climate change. No significant differences were found between groups, *p* > 0.143. Values below 5% were not specified.

Engagement in summer mountain sport activity when confronted with consequences of climate change was rated similarly between groups, χ^2^ (4, *N* > 243) <6.87, *p* > 0.143 (Figure 3). Rockfall/Rock collapse was rated as the most important consequence for a *reduced* regularity of conducting summer mountain sport. Melting of snow earlier in the season as the most important consequence for an *increased* regularity of conducting summer mountain sport. The other consequences of climate change were predominantly rated (>62%) as not concerning the regularity of summer mountain sport.

### Regression Analyses

**Figure 4:**
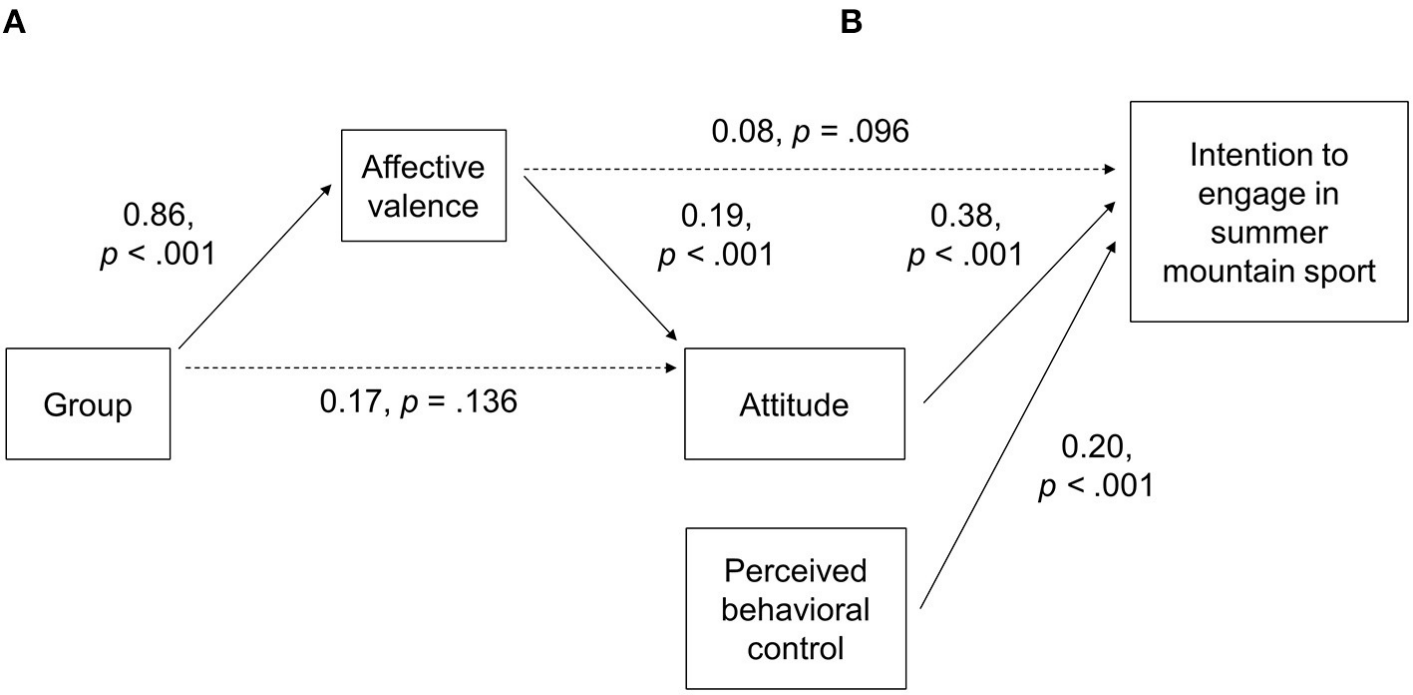
Regression analysis of **(A)** attitude as an outcome of group allocation or affective valence and **(B)** hierarchical regression analysis of intention to engage in summer mountain sport as an outcome of attitude, perceived behavioral control (model 1), and affective valence (model 2). Path coefficients show standardized regression coefficients. Solid lines indicate statistically significant coefficients; dashed lines indicate non-significant coefficients.

Group allocation was not significantly related to attitude (Figure 4A). The significant coefficient of group allocation on affective valence reflects the result of higher affective valence in the CCU group compared to the CCA group. A higher affective valence was significantly associated with a higher attitude toward summer mountain sport.

Both attitude toward summer mountain sport and perceived behavioral control was significantly positively associated with intention to engage in summer mountain sport (Figure 4B). *R*^2^ of the multiple model of step 1 was 24.2%. The inclusion of affective valence to the model (step 2) did not significantly improve *R*^2^ (*R*^2^ of step 2: 24.9%), *p* = 0.096.

## Discussion

Aim 1 of the present study was to analyze behaviorally relevant variables for the engagement in recreational summer mountain sport activities in a climate change affected scenario. The present results indicate that the intention, attitude, and perceived behavioral control are similar in comparison to a climate change unaffected scenario. Anticipated affective responses valence and activation were rated lower in the climate change affected scenario. Aim 2 focused on the role of anticipated affective valence on the intention to engage in mountain sport in the context of TPB. Anticipated affective valence did not significantly improve the TPB model. However, higher values in affective valence were associated with a higher attitude toward summer mountain sport.

### Similar Intention to Engage in Summer Mountain Sport Activities

The intention to engage in summer mountain sport activities was rated high, with a mean value of the total sample close to the maximum. This result is desirable from a health behavior perspective, since the probability to actually engage in mountain sport activities is largely dependent on the intention (36). Since physical activity is seen as an opportunity to reach multiple sustainable development goals, engagement in mountain sport activities might have an impact even beyond physiological and psychological benefits (37). Given the similarly high values between the group being exposed to a climate change affected scenario and the group being exposed to a climate change unaffected scenario, an altered intention (and potentially behavior), due to climate change effects in mountainous environments shown by the pictures, is not expected. This result contrasts our findings in winter sport, where an identical design was used and a lower intention in the climate change affected scenario was found (16). This discrepancy between summer and winter mountain sport might be explained by the following: Mountain sport both in the winter (38) and in the summer season (23, 26) consists a variety of different activities. However, in winter sport, a lack of snow/glacier retreat is perceived as a thread to being able to conduct the activities (39). Consistent to the Protection Motivation Theory people show a higher likelihood to change behavior if the personal threat appraisal is high (40). Although it is possible to conduct winter sport on artificial snow, the preference for sufficient natural snow conditions is higher and people tend to travel farther to destinations with higher probability for natural snow (41). In contrast, in most of the mountain sports conducted in the summer season, e.g., mountain hiking, mountain biking, or sport climbing, a lack of snow or glacier retreat is not perceived as a thread, but may be perceived as an opportunity to conduct the activity longer in the season or at an higher altitude (42). Conformingly, the results of the present study on future summer mountain sport engagement in case of large glacier shrinkage suggest an increased frequency in between 9 and 23% of the sample. Furthermore, melting of snow earlier in the season was rated as a consequence of climate change to increase the regularity of summer mountain sport. It has to be noted that melting of snow earlier in the season may only affect the timing of summer mountain sport activities instead of the regularity. Not all consequences of climate change were asked in the present study. Furthermore, not all consequences of climate change affect summer mountain sport activities to the same extent. Although dependent on the exact location, where the activity is conducted, engagement in mountain hiking and mountain biking might be least (negatively) affected by the consequences of climate change. Consequences like a higher number of days with thermally comfortable conditions and a higher number of days with sunshine or little precipitation may even increase engagement in these activities (43). This is also expectable for fixed-rope climbing, outdoor sport climbing, and outdoor bouldering, although increased rockfall earlier in the season caused by the thaw-freezing cycle (44) may be perceived as a risk and in consequence reduce the engagement in these activities. All consequences connected to glacial changes are expected to have little impact on the aforementioned summer mountain sport activities, but the highest negative impact on multipitch climbing and mountaineering in glacial terrain with some itineraries being not climbable anymore (27).

In the present study, we presented pictures showing glacier retreat. Glacier retreat as a consequence of climate change was chosen based on the consideration that it is less connected to seasonal changes compared to other consequences of climate change (e.g., earlier melting of snow, higher snow line). Pictures showing a higher snow line might be more perceived as a sign of summer and not as a consequence of climate change. Another consideration was that the consequences of climate change should be visible in pictures, which is difficult to achieve for other changes due to climate change, e.g., rockfall (27) or a higher number of days with sunshine (43). Given the differentiated effects of consequences of climate change on summer mountain sport activities, it should be considered that glacier retreat affects some mountain sport activities more (multipitch climbing and mountaineering in glacial terrain) than others (e.g., mountain hiking or mountain biking).

In mountaineering or (in parts) alpine multipitch climbing, climate change can pose severe effects on the environment, where the activity takes place (20). Since these effects include rock collapse, which is connected with a total destruction of the mountaineering itineraries (27), effects of climate change in mountaineering might be—similar to winter sport (16)—perceived as a threat to being able to conduct the activity. However, in the subsample of mountaineers, like in the total sample, no significant group differences in the TPB variables were found. Obviously, the personal threat appraisal for mountaineering was too low to result in behavior intention changes (40). Although there were some indications that mountaineering is considered differently to other summer mountain sport activities, as mountaineering was the activity with the least percentage of the same frequency, there were still more than 20% who stated an increase of conducting mountaineering in case of large glacier shrinkage. We speculate that glacier shrinkage leads more to a change in the selection of the time in the season or in the choice of mountaineering itineraries, as shown in the Mont Blanc region (27), instead of a change in general mountaineering behavior.

The risks connected to environmental changes due to climate change should also be considered in the behavioral context. The present results on engagement in summer mountain sport activity indicate that when confronted with some of these risks, participants tend to state a lower regularity of engaging in summer mountain sport, e.g., rockfall or changes in crevasses. However, the answers did not significantly differ between groups. We acknowledge that asking the participants for their engagement when being confronted with the consequence of climate change can only provide a first insight since the selection of the consequences was based on the effects of climate change in mountaineering (27). Rockfall/rock collapse was rated as the most important consequence to decrease regularity in mountain summer sport activities. Indeed, rockfall is among the most important risk factors for fatalities in mountaineering (23).

### Affective Valence in the Behavioral Context

Affective experiences made in physical activity bouts were repeatedly shown to be predictive of future physical activity (10). Specifically, more positive affective valence during physical activity bouts were associated with higher probability to engage in physical activity in the future (9). The affect-physical activity behavior relation inspires to study affective responses in the physical activity context (15, 45–49). Since the TPB variables can be considered as a “planned” or cognitively oriented approach, our intention was to integrate affective states in the context of summer mountain sport activities. The present data confirmed parts of the TPB model (4) with perceived behavioral control and attitude as significant positively related predictors of intention. However, the extension through anticipated affective valence did not significantly improve the model and added <1% of explained variance to the model. Therefore, a direct influence of affective valence on intention seems less likely or marginally. The present finding partly contrasts existing findings outside of the mountain sport context: Kwan and Bryan (50) showed a positive association of positive affect during a 30-min bout of treadmill exercise and intention to engage in physical activity 3 months later. It has to be noted that the authors used the slope in positive affect to predict intention; a procedure, which takes into account variations in affect at the baseline (50). Given the cross-sectional design of the present study, this approach was not used and might be an explanation for the discrepancy of the results and for the replication of our finding in winter sport, where we did also not assess baseline affect (16). Another explanation might be the time between the assessment of affect and attitude. While Kwan and Bryan (50) assessed affect to the physical activity bout 3 months prior to the intention to engage in physical activity, only minutes were between the assessment of affect and intention in the present study. It might be assumed that non-cognitive effects on intention might take longer to occur and might be found after repeated experiences of reduced valence.

It is important to mention, that the finding of a less likely influence of affective valence on intention does not mean that affective variables should be neglected in physical activity behavior. In the present study, higher values in affective valence were associated with a higher attitude toward summer mountain sport. This association confirms previous findings (16, 50, 51) and suggests that the collection of affective data is important in the attitude toward behavior. Thus, and given a potential longer-term behavior changes due to affective valence mentioned above, non-cognitive affective variables should be considered when trying to engage people in physical activity behavior.

### Limitations and Strengths

When interpreting the results of the present study, the following limitations should be taken into account. Firstly, we did not assess actual physical activity behavior, but intention to engage in summer mountain sport. Although the intention to engage in a certain behavior is connected to the actual behavior (36), the present study is limited to the intention. Secondly, the present sample was recruited in the Eastern Alps only. Given the differences in geographical characteristics (e.g., altitude of the summits) and in mountain sport activities, the present results cannot be generalized to the Western Alps or other mountainous regions. Connected to this aspect, the risk perception of the present sample was relatively high (33). However, given the similarity between groups in the present sample risk, we assume that group differences in mountain sport practitioners being less sensitive to risk perception of climate change cannot be expected. Thirdly, the choice of pictures displaying the scenarios is crucial and possibly introduces a large inter-individual variability in the participants' associations with the pictures. Although the selection of the pictures was attained after an intense internal review including a rating and discussion process, pictures showing other changes connected to climate change might produce different results. Future research might consider using other approaches for the choice of pictures. These approaches include (a) conducting a pre-study to find most appropriate pictures illustrating climate change, which are compatible with the type of physical activity conducted by the persons (e.g., difference between hiking at high altitude vs. mountain biking in lower altitudes), (b) providing a selection phase within the questionnaire, where participants are asked on several pictures to select the one that best illustrates climate change, which is subsequently used in the questionnaire, (c) using pictures of an existing database where relevance to climate changes was already conducted (52). Fourthly, the variety of summer mountain sport activities makes the selection of consequences of climate change difficult since the effects of climate change depend on the type of activity. In the same context. the specification of mountaineers in the present study cannot be seen as a formal definition. The term mountaineers in the present study was used for a subgroup of the sample participating in mountaineering. Future studies may account for this by focusing on a subgroup of a specific form of mountain sport practitioners only.

Strengths of the study include a wider recruitment over an Alpine association not limited to students/university employees, a reliable randomization, and well-comparable scenarios. In contrast to our previous study (16), we chose to use one identical picture for both groups. To control other potentially influencing factors in the scenarios (e.g., different summits, people, environmental characteristics), the only differences between the scenarios was the year and the second picture indicating the glacier retreat.

## Conclusion

In mountain sport activities conducted in the summer season, intention to engage in mountain sport was similar in participants being exposed to the climate change affected and unaffected scenarios. In contrast to existing findings in winter mountain sport activities, intention to engage in summer mountain sport seems to be less affected by scenarios displaying climate change. Therefore, a reduced engagement in mountain sport activities due to climate change seems less likely in summer compared to winter season. However, the variety of summer mountain sport activities should be considered. The present findings reflect summer mountain sport in general without focusing on a single summer mountain sport activity, although glacier shrinkage might affect the engagement in the summer mountain sport activities differently. We did not find differences in the subsample of mountaineers, who are expected to be most affected by glacier shrinkage; however, differentiated findings across different activities of summer mountain sport cannot be excluded.

Furthermore, in the presence of clear signs of climate change in the environment, summer mountain sport might be associated with a reduced affective valence since affective valence was significantly lower in the climate change affected scenario. Although we did not find a direct effect of affective valence on intention, a reduced affective valence might dampen the attitude toward mountain sport, what might become behaviorally relevant in the longer term by repeated experiences of reduced valence during summer mountain sport activities. Future research might focus on practitioners of summer mountain sport activities that are most affected by climate change, e.g., mountaineering.

## Data Availability Statement

The raw data supporting the conclusions of this article will be made available by the authors, without undue reservation.

## Ethics Statement

The studies involving human participants were reviewed and approved by the Board for Ethical Questions in Science of the University of Innsbruck in accordance with the Declaration of Helsinki (#44/2021, date: 21.06.2021). The Ethics Committee waived the requirement of written informed consent for participation.

## Author Contributions

MN, AF, and MK contributed to conception and design of the study. MN and AF collected the data. AF wrote a first draft of the introduction. MN analyzed the data and drafted the rest of the manuscript. All authors reviewed the manuscript and approved the submitted version.

## Conflict of Interest

The authors declare that the research was conducted in the absence of any commercial or financial relationships that could be construed as a potential conflict of interest. The reviewer RS declared a shared affiliation with the authors MN, AF, and MK to the handling editor at the time of review.

## Publisher's Note

All claims expressed in this article are solely those of the authors and do not necessarily represent those of their affiliated organizations, or those of the publisher, the editors and the reviewers. Any product that may be evaluated in this article, or claim that may be made by its manufacturer, is not guaranteed or endorsed by the publisher.

## References

[1] Guthold R Stevens GA Riley LM Bull FC. Worldwide trends in insufficient physical activity from 2001 to 2016: a pooled analysis of 358 population-based surveys with 1·9 million participants. Lancet Global Health. (2018) 6:e1077–86. 10.1016/S2214-109X(18)30357-7 30193830

[2] Pedersen BK Saltin B. Exercise as medicine - evidence for prescribing exercise as therapy in 26 different chronic diseases. Scand J Med Sci Sports. (2015) 3(Suppl. 25):1–72. 10.1111/sms.12581 26606383

[3] World Health Organisation. Fact Sheet Physical Activity. Available online at:https://www.who.int/news-room/fact-sheets/detail/physical-activity (accessed March 22, 2021).

[4] Ajzen I. The theory of planned behavior. Organ Behav Hum Decis Process. (1991) 50:179–211. 10.1016/0749-5978(91)90020-T

[5] Ekkekakis P Petruzzello SJ. Analysis of the affect measurement conundrum in exercise psychology: IV. A conceptual case for the affect circumplex. Psychol Sport Exercise. (2002) 3:35–63. 10.1016/S1469-0292(01)00028-0

[6] Fishbein M Ajzen I. Predicting and Changing Behavior: The Reasoned Action Approach. New York, NY: Psychology Press (2010).

[7] Hassandra M Vlachopoulos SP Kosmidou E Hatzigeorgiadis A Goudas M Theodorakis Y. Predicting students' intention to smoke by theory of planned behaviour variables and parental influences across school grade levels. Psychol Health. (2011) 26:1241–58. 10.1080/08870446.2011.605137 21834644

[8] Norman P Cooper Y. The theory of planned behaviour and breast self-examination: assessing the impact of past behaviour, context stability and habit strength. Psychol Health. (2011) 26:1156–72. 10.1080/08870446.2010.481718 21391130

[9] Williams DM Dunsiger S Ciccolo JT Lewis BA Albrecht AE Marcus BH. Acute affective response to a moderate-intensity exercise stimulus predicts physical activity participation 6 and 12 months later. Psychol Sport Exerc. (2008) 9:231–45. 10.1016/j.psychsport.2007.04.002 18496608 PMC2390920

[10] Rhodes RE Kates A. Can the affective response to exercise predict future motives and physical activity behavior? A systematic review of published evidence. Ann Behav Med. (2015) 49:715–31. 10.1007/s12160-015-9704-5 25921307

[11] Ekkekakis P Hall EE Petruzzello SJ. Some like it vigorous: measuring individual differences in the preference for and tolerance of exercise intensity. J Sport and Exercise Psychol. (2005) 27:350–74. 10.1123/jsep.27.3.350

[12] Shanahan DF Franco L Lin BB Gaston KJ Fuller RA. The benefits of natural environments for physical activity. Sports Med. (2016) 46:989–95. 10.1007/s40279-016-0502-4 26886475

[13] Flowers EP Freeman P Gladwell VF. A cross-sectional study examining predictors of visit frequency to local green space and the impact this has on physical activity levels. BMC Public Health. (2016) 16:420. 10.1186/s12889-016-3050-9 27207300 PMC4875629

[14] Thompson Coon J Boddy K Stein K Whear R Barton J Depledge MH. Does participating in physical activity in outdoor natural environments have a greater effect on physical and mental wellbeing than physical activity indoors? A systematic review. Environ Sci Technol. (2011) 45:1761–72. 10.1021/es102947t 21291246

[15] Niedermeier M Einwanger J Hartl A Kopp M. Affective responses in mountain hiking-A randomized crossover trial focusing on differences between indoor and outdoor activity. PLoS ONE. (2017) 12:e0177719. 10.1371/journal.pone.0177719 28520774 PMC5433751

[16] Frühauf A Niedermeier M Kopp M. Intention to engage in winter sport in climate change affected environments. Front Public Health. (2020) 8:598297. 10.3389/fpubh.2020.598297 33392137 PMC7775576

[17] Rhodes RE Dickau L. Moderators of the intention-behaviour relationship in the physical activity domain: a systematic review. Br J Sports Med. (2013) 47:215–25. 10.1136/bjsports-2011-090411 22278998

[18] Sommer C Malz P Seehaus TC Lippl S Zemp M Braun MH. Rapid glacier retreat and downwasting throughout the European Alps in the early 21(st) century. Nat Commun. (2020) 11:3209. 10.1038/s41467-020-16818-0 32587270 PMC7316704

[19] Grabherr G Gottfried M Pauli H. Climate change impacts in alpine environments. Geography Compass. (2010) 4:1133–1153. 10.1111/j.1749-8198.2010.00356.x

[20] Gobiet A Kotlarski S Beniston M Heinrich G Rajczak J Stoffel M. 21st century climate change in the European Alps–a review. Sci Total Environ. (2014) 493:1138–51. 10.1016/j.scitotenv.2013.07.050 23953405

[21] Statistik Austria. Auslastung Der Betten (in %) Für Die Winter- Und Sommersaison Von 2004 bis 2020 Nach Bundesländern. (2020). Available online at: https://www.statistik.at/web_de/statistiken/wirtschaft/tourismus/beherbergung/betriebe_betten/index.html (accessed November 1, 2021).

[22] Askew AE Bowker JM. Impacts of climate change on outdoor recreation participation: outlook to 2060. J Park Recreat Admin. (2018) 36:97–120. 10.18666/JPRA-2018-V36-I2-8316

[23] Gatterer H Niedermeier M Pocecco E Frühauf A Faulhaber M Menz V. Mortality in different mountain sports activities primarily practiced in the summer season—a narrative review. Int J Environ Res Public Health. (2019) 16:3920. 10.3390/ijerph16203920 31618960 PMC6843304

[24] Ajzen I. Constructing a TPB Questionnaire: Conceptual and Methodological Considerations. Available online at: https://pdfs.semanticscholar.org/0574/b20bd58130dd5a961f1a2db10fd1fcbae95d.pdf (accessed July 13, 2020).

[25] Pickering CM Castley JG Burtt M. Skiing less often in a warmer world: attitudes of tourists to climate change in an australian ski resort. Geograp Res. (2010) 48:137–147. 10.1111/j.1745-5871.2009.00614.x

[26] Niedermeier M Hartl A Kopp M. Prevalence of mental health problems and factors associated with psychological distress in mountain exercisers: a cross-sectional study in Austria. Front Psychol. (2017) 8:1237. 10.3389/fpsyg.2017.01237 28775701 PMC5517492

[27] Mourey J Marcuzzi M Ravanel L Pallandre F. Effects of climate change on high Alpine mountain environments: Evolution of mountaineering routes in the Mont Blanc massif (Western Alps) over half a century. Arctic Antarctic Alpine Res. (2019) 51:176–89. 10.1080/15230430.2019.1612216

[28] Russell JA. A circumplex model of affect. J Person Soc Psychol. (1980) 39:1161–78. 10.1037/h0077714

[29] Maibach M Niedermeier M Sudeck G Kopp M. Erfassung unmittelbarer affektiver Reaktionen auf körperliche Aktivität [in German]. Zeitschrift Sportpsychol. (2020) 27:4–12. 10.1026/1612-5010/a000291

[30] Hardy CJ Rejeski WJ. Not what, but how one feels: the measurement of affect during exercise. J Sport Exercise Psychol. (1989) 11:304–17. 10.1123/jsep.11.3.304

[31] Svebak S Murgatroyd S. Metamotivational dominance: a multimethod validation of reversal theory constructs. J Person Soc Psychol. (1985) 48:107. 10.1037/0022-3514.48.1.10718835411

[32] Johansson G Westerterp K. Assessment of the physical activity level with two questions: validation with doubly labeled water. Int J Obesity 2005. (2008) 32:1031–3. 10.1038/ijo.2008.42 18392036

[33] Leiserowitz A. Climate change risk perception and policy preferences: the role of affect, imagery, and values. Climatic Change. (2006) 77:45–72. 10.1007/s10584-006-9059-9

[34] Cohen J. Statistical Power Analysis for the Behavioral Sciences. Hillsdale: Lawrence Erlbaum Associates (1988).

[35] The jamovi project. Jamovi (Version 2.0): Computer Software. (2020). Available online at: https://www.jamovi.org (accessed October 1, 2021).

[36] Hagger MS Chatzisarantis NLD Biddle SJH. A meta-analytic review of the theories of reasoned action and planned behavior in physical activity: predictive validity and the contribution of additional variables. J Sport Exercise Psychol. (2002) 24:29. 10.1123/jsep.24.1.3

[37] Salvo D Garcia L Reis RS Stankov I Goel R Schipperijn J. Physical activity promotion and the united nations sustainable development goals: building synergies to maximize impact. J Phys Act Health. (2021) 18:1163–80. 10.1123/jpah.2021-0413 34257157

[38] Niedermeier M Gatterer H Pocecco E Frühauf A Faulhaber M Menz V. Mortality in different mountain sports activities primarily practiced in the winter season—a narrative review. Int J Environ Res Public Health. (2019) 17:259. 10.3390/ijerph17010259 31905912 PMC6981978

[39] Elsasser H Bürki R. Climate change as a threat to tourism in the Alps. Climate Res. (2002) 20:253–7. 10.3354/cr020253

[40] Floyd DL Prentice-Dunn S Rogers RW. A meta-analysis of research on protection motivation theory. J Appl Soc Psychol. (2000) 30:407–29. 10.1111/j.1559-1816.2000.tb02323.x

[41] Unbehaun W Pröbstl U Haider W. Trends in winter sport tourism: challenges for the future. Tourism Rev. (2008) 63:36–47. 10.1108/16605370810861035

[42] Pröbstl-Haider U Haider W Wirth V Beardmore B. Will climate change increase the attractiveness of summer destinations in the European Alps? A survey of German tourists. J Outdoor Recreat Tourism. (2015) 11:44–57. 10.1016/j.jort.2015.07.003

[43] Pröbstl-Haider U Hödl C Ginner K Borgwardt F. Climate change: impacts on outdoor activities in the summer and shoulder seasons. J Outdoor Recreat Tourism. (2021) 34:100344. 10.1016/j.jort.2020.100344

[44] Lack DA Sheets AL Entin JM Christenson DC. Rock climbing rescues: causes, injuries, and trends in boulder county, colorado. Wilderness Environ Med. (2012) 23:223–30. 10.1016/j.wem.2012.04.002 22727678

[45] Ekkekakis P. Pleasure and displeasure from the body: perspectives from exercise. Cognit Emotion. (2003) 17:213–39. 10.1080/02699930302292 29715726

[46] Sudeck G Schmid J Conzelmann A. Exercise experiences and changes in affective attitude: direct and indirect effects of in situ measurements of experiences. Front Psychol. (2016) 7:900. 10.3389/fpsyg.2016.00900 27378992 PMC4909746

[47] Niedermeier M Grafetstatter C Kopp M Huber D Mayr M Pichler C. The role of anthropogenic elements in the environment for affective states and cortisol concentration in mountain hiking-a crossover trial. Int J Environ Res Public Health. (2019) 16:290. 10.3390/ijerph16020290 30669640 PMC6352183

[48] Niedermeier M Weiss EM Steidl-Müller L Burtscher M Kopp M. Acute effects of a short bout of physical activity on cognitive function in sport students. Int J Environ Res Public Health. (2020) 17:3678. 10.3390/ijerph17103678 32456170 PMC7277588

[49] Dierkes K Mattioni Maturana F Rösel I Martus P Nieß AM Thiel A. Different endurance exercise modalities, different affective response: a within-subject study. Front Psychol. (2021) 12:686661. 10.3389/fpsyg.2021.686661 34484040 PMC8411706

[50] Kwan BM Bryan AD. Affective response to exercise as a component of exercise motivation: attitudes, norms, self-efficacy, and temporal stability of intentions. Psychol Sport Exerc. (2010) 11:71–79. 10.1016/j.psychsport.2009.05.010 20161385 PMC2782828

[51] Catellier JRA Yang ZJ. The role of affect in the decision to exercise: does being happy lead to a more active lifestyle? Psychol Sport Exercise. (2013) 14:275–82. 10.1016/j.psychsport.2012.11.006

[52] Lehman B Thompson J Davis S Carlson JM. Affective images of climate change. Front Psychol. (2019) 10:960. 10.3389/fpsyg.2019.00960 31156493 PMC6529642

