# Supplementary material for: Intention to Engage in Mountain Sport During the Summer Season in Climate Change Affected Environments
Source: Front Public Health. 2022 Jul 7;10:828405. doi: 10.3389/fpubh.2022.828405 (PMC9300943; doi:10.3389/fpubh.2022.828405)
Supplement: Supplementary file 1 [file Data_Sheet_1.PDF]

Recruitment information

Dear mountain sport enthusiasts!

As part of a research project of the Department of Sport Science at the University of Innsbruck, we are looking for mountain sport enthusiasts, who have engaged in mountain sport activities (e.g., mountain hiking, mountain biking, or alpine climbing) in the summer season 2021 that is now ending. If you are at least 18 years old, we would appreciate if you could take about 10 minutes to answer the questionnaire below.

The aim of the research project is to assess the motivation of practicing mountain sport activities in summer in certain scenarios. The scenarios are represented by pictures.

The questionnaire is available at the following link:

[Link to the questionnaire]

Thank you for the interest!

Initial information at the start of the questionnaire

Thank you for your interest in our research project. In the following, you will find - in addition to general questions about yourself - questions that relate to your motivation to engage in mountain sport activities in the summer season in certain scenarios. The scenarios are represented by pictures.

Answering the questionnaire will take about 10 minutes. Please take the time to answer the questions carefully and truthfully.

[Privacy policy statement]

Thank you for your participation!

The following questions refer to your **mountain sport activities in the 2021 summer season** (since the month of May).

In the 2021 summer season, how many times on average did you participate in mountain sport activities in a regular month?

___________Number of mountain sport activities in summer per month

[Please compare footnote [1]]

During the 2021 summer season, which of the following mountain sport activities did you engage in?

	1: Never	2	3	4	5	6	7: Regul arly
Mountain hiking on wider trails, valley paths or forest paths	☐	☐	☐	☐	☐	☐	☐
Mountain hiking on narrower mountain paths or climbs	☐	☐	☐	☐	☐	☐	☐
Mountain biking	☐	☐	☐	☐	☐	☐	☐
Fixed-rope climbing (via ferrata)	☐	☐	☐	☐	☐	☐	☐
Outdoor sport climbing	☐	☐	☐	☐	☐	☐	☐
Outdoor bouldering	☐	☐	☐	☐	☐	☐	☐
Alpine multipitch climbing	☐	☐	☐	☐	☐	☐	☐
Mountaineering (glacier)	☐	☐	☐	☐	☐	☐	☐

Are there any other mountain sport activities you engaged in during the 2021 summer season that were not mentioned?

____

[1] Given the justified comments of one of the Reviewers, we recommend for future research in this field to adapt:
a) The position of this question in the questionnaire, i.e., to move this question after “Are there any other mountain sport activities you engaged in during the 2021 summer season that were not mentioned?”
b) The formulation of this question: “In the 2021 summer season, how many times on average did you participate in mountain sport activities per month?”

The following questions refer to your **general physical activity behavior** (and not exclusively to mountain sport activities).

Describe your physical activity at work (even work at home, sick leave at home and studying, for instance in a university).

1: Very light, e.g., sitting at the computer most of the day or sitting at a desk	2: Light, e.g., light industrial work, sales or office work that comprises light activities	3: Moderate, e.g., cleaning, staffing at kitchen or delivering mail on foot or by bicycle	4: Heavy, e.g., heavy industrial work, construction work or farming
☐	☐	☐	☐

Describe your physical activity at leisure time. If the activities vary between summer and winter, try to give a mean estimate

1: Very light: almost no activity at all	2: Light, e.g., walking, nonstrenuous cycling or gardening approximately once a week	3: Moderate: regular activity at least once a week, e.g., walking, bicycling, or gardening or walking to work 10–30 min/day	4: Active: regular activities more than once a week, e.g., intense walking or bicycling or sports	5: Very active: strenuous activities several times a week
☐	☐	☐	☐	☐

Below we ask you for a few personal details.

Please indicate your sex. Female ☐ Male ☐

Please indicate your age.

_______________________ years

Please indicate your height.

_______________________ cm

Please indicate your weight.

_______________________ kg

Please select the appropriate answer from the following options.

	1: Not at all	2	3	4: Very
How concerned are you about global warming?	☐	☐	☐	☐
How likely do you think it is that worldwide, many people's standard of living will decrease?	☐	☐	☐	☐
How likely do you think it is that your standard of living will decrease?	☐	☐	☐	☐

[Randomization to either climate change unaffected (CCU) or climate change affected (CCA) picture(s)]

In the following you will now see the identical picture(s) repeatedly.
We ask you to first observe the picture(s) and then answer the questions below in the imagination of engaging in mountain sport activities in the environment seen in the picture(s).

[either CCU or CCA picture(s)]

Estimate here how **good or bad** you feel when you imagine yourself practicing your mountain sport in the picture(s) shown.

-5: very bad	-4	-3: bad	-2	-1: fairly bad	0: neut ral	1: fairly good	2	3: good	4	5: very good
☐	☐	☐	☐	☐	☐	☐	☐	☐	☐	☐

Estimate here how **aroused** you actually feel when you imagine yourself practicing your mountain sport in the picture(s) shown. By “arousal” we meant how “worked-up” you feel. You might experience high arousal in one of a variety of ways, for example as excitement or anxiety or anger. Low arousal might also be experienced by you in one of a number of different ways, for example as relaxation or boredom or calmness.

1: low arousal	2	3	4	5	6: high arousal
☐	☐	☐	☐	☐	☐

Please observe the picture(s) again and then answer the questions below in the imagination of engaging in mountain sport activities in the environment seen in the picture(s).

[either CCU or CCA picture(s)]

I intend to participate regularly in mountain sport activities in the upcoming summer season.

1: extremely unlikely	2	3	4	5	6	7: extremely likely
☐	☐	☐	☐	☐	☐	☐

I will try to engage in mountain sport activities in the upcoming summer season on a regular basis.

1: definitely false	2	3	4	5	6	7: definitely true
☐	☐	☐	☐	☐	☐	☐

I am planning to engage in mountain sport activities in the upcoming summer season on a regular basis.

1: strongly disagree	2	3	4	5	6	7: strongly agree
☐	☐	☐	☐	☐	☐	☐

For me, a regular participation in mountain sport activities (at least once a week) in the upcoming summer season is:

	1	2	3	4	5	6	7	
Harmful	☐	☐	☐	☐	☐	☐	☐	Beneficial
Pleasant	☐	☐	☐	☐	☐	☐	☐	Unpleasant
Good	☐	☐	☐	☐	☐	☐	☐	Bad
Worthless	☐	☐	☐	☐	☐	☐	☐	Valuable
Enjoyable	☐	☐	☐	☐	☐	☐	☐	Not enjoyable

Please observe the picture(s) again and then answer the questions below in the imagination of engaging in mountain sport activities in the environment seen in the picture(s).

[either CCU or CCA picture(s)]

I am confident that I will be able to regularly conduct mountain sport activities in the coming summer season.

1: disagree	2	3	4	5	6	7: agree
☐	☐	☐	☐	☐	☐	☐

It is up to me to engage in mountain sport activities in the coming summer season on a regular basis.

1: disagree	2	3	4	5	6	7: agree
☐	☐	☐	☐	☐	☐	☐

I am convinced that I will engage in mountain sport activities regularly in the upcoming summer season.

1: disagree	2	3	4	5	6	7: agree
☐	☐	☐	☐	☐	☐	☐

I can influence whether I engage in mountain sport activities regularly in the coming summer season.

1: disagree	2	3	4	5	6	7: agree
☐	☐	☐	☐	☐	☐	☐

Assuming that the next five winters had major impact on the melting of glaciers, which of the answers would apply to you and the mountain sport activity? If you do not engage in the mountain sport activities, please use the "No answer" option.

	1: Quit mountain sport	2: Conduct mountain sport further away	3: Conduct mountain sport less often than now	4: Conduct mountain sport in the same frequency as now	5: Conduct mountain sport more often than now	No answer
Mountain hiking on wider trails, valley paths or forest paths	☐	☐	☐	☐	☐	☐
Mountain hiking on narrower mountain paths or climbs	☐	☐	☐	☐	☐	☐
Mountain biking	☐	☐	☐	☐	☐	☐
Fixed-rope climbing (via ferrata)	☐	☐	☐	☐	☐	☐
Outdoor sport climbing	☐	☐	☐	☐	☐	☐
Outdoor bouldering	☐	☐	☐	☐	☐	☐
Alpine multipitch climbing	☐	☐	☐	☐	☐	☐
Mountaineering (glacier)	☐	☐	☐	☐	☐	☐

Which consequences of climate change stated below affect the regularity of your mountain sport activities in the summer?

This question aims at all mountain sport activities in summer regardless of the different types of activity (mountain hiking, mountain biking, etc.).

	1: Quit mountain sport	2: Less regularly	3: Does not concern	4: More regularly	5: Much more regularly
Rockfall/Rock collapse	☐	☐	☐	☐	☐
Glacial shrinkage	☐	☐	☐	☐	☐
Melting of snow earlier in the season	☐	☐	☐	☐	☐
Changes in glacier moraines	☐	☐	☐	☐	☐
Changes in crevasses/Bergschrunds	☐	☐	☐	☐	☐
Bare ice on the glaciers	☐	☐	☐	☐	☐
Slope angle increases	☐	☐	☐	☐	☐

Are there any other consequences of climate change that affect the regularity of your mountain sport activities in summer that were not mentioned?

____

The questionnaire is now finished, and you can close the browser. Thank you for your cooperation.
